# ‘Clustering’ SIRPα into the Plasma Membrane Lipid Microdomains Is Required for Activated Monocytes and Macrophages to Mediate Effective Cell Surface Interactions with CD47 

**DOI:** 10.1371/journal.pone.0077615

**Published:** 2013-10-15

**Authors:** Binh Ha, Zhiyuan Lv, Zhen Bian, Xiugen Zhang, Aarti Mishra, Yuan Liu

**Affiliations:** 1 Program of Cellular Biology and Immunology, Center of Inflammation, Immunity and Infection, Georgia State University, Atlanta, Georgia, United States of America; 2 School of Life Sciences, Nanjing University, Nanjing, Jiangsu, China; UC San Diego, United States of America

## Abstract

SIRPα, an ITIMs-containing signaling receptor, negatively regulates leukocyte responses through extracellular interactions with CD47. However, the dynamics of SIRPα-CD47 interactions on the cell surface and the governing mechanisms remain unclear. Here we report that while the purified SIRPα binds to CD47 and that SIRPα is expressed on monocytes and monocytic THP-1 or U937, these SIRPα are ineffective to mediate cell binding to immobilized CD47. However, cell binding to CD47 is significantly enhanced when monocytes transmigrating across endothelia, or being differentiated into macrophages. Cell surface labeling reveals SIRPα to be diffused on naïve monocytes but highly clustered on transmigrated monocytes and macrophages. Protein crosslink and equilibrium centrifugation confirm that SIRPα in the latter cells forms oligomerized complexes resulting in increased avidity for CD47 binding. Furthermore, formation of SIRPα complexes/clusters requires the plasma membrane ‘lipid rafts’ and the activity of Src family kinase during macrophage differentiation. These results together suggest that ‘clustering’ SIRPα into plasma membrane microdomains is essential for activated monocytes and macrophages to effectively interact with CD47 and initiate intracellular signaling.

## Introduction

Signal regulatory protein α (SIRPα) (also known as SHPS1, CD172A, or P84) is a cell surface receptor-like signaling protein predominantly expressed in myeloid leukocytes including granulocytes, monocytes, macrophages and dendritic cells [1,2]. The expression of SIRPα is also reported in other specific types of cells such as neurons [3] and podocytes in the kidney [4]. As a typical type-1 transmembrane protein, SIRPα functions tightly correlate with its structure, which contains an extracellular domain with a highly interactive IgV-like loop and a cytoplasmic domain containing two immunoreceptor tyrosine-based inhibitory motifs (ITIMs) that are essential for signaling [5]. SIRPα has been reported to be crucial for leukocyte functions, including monocyte and neutrophil (PMN) chemotactic transmigration [6,7], macrophage phagocytosis [8], macrophage fusion [9], dendritic cell antigen presentation [10], and other possible cellular functions such as neuronal axonal growth and synapse formation [3] and kidney filtration [4]. The regulation of all these cellular functions by SIRPα has been attributed to its extracellular binding interactions with the ligand CD47, another important cell surface protein broadly expressed on most cells, and to the SIRPα ITIMs-mediated intracellular signaling. It has been suggested that the extracellular binding of SIRPα to CD47 triggers tyrosine phosphorylation in its cytoplasmic ITIMs, resulting in the association of the SH2 domain-containing protein tyrosine phosphatase (SHP-1 or 2), which consequently initiates negative signaling events leading to the inhibition of cell function [11-16]. Studies of macrophage phagocytosis indicate that ligation of macrophage SIRPα by CD47 expressed on the encountered cells prohibits macrophage phagocytosis, whereas failure of SIRPα engagement by CD47, or deficiency of SIRPα ITIM-mediated signaling, promotes macrophage engulfment of the cell [2,8,17-22]. It has also been suggested that increased CD47 expression on cancer cells serves as a crucial mechanism for the evasion of immune clearance [23,24].

The evidence found for high affinity binding between the extracellular domains of SIRPα and CD47 is convincing. Experiments using recombinant SIRPα and CD47 extracellular domains as well as transfected cells that overexpress the full-length SIRPα or CD47 protein on the cell surface [7,25-27] have demonstrated efficient binding between the two proteins. Despite being expressed by different cell types, we previously observed that CD47 purified from RBCs, leukocytes, spleen cells, fibroblasts and epithelial cells all directly bound to SIRPα [7]. Key amino acid residues in the SIRPα extracellular IgV-like loop, which mediates binding to CD47, have also been identified [26]. However, most of these experiments confirming SIRPα-CD47 binding were performed in acellular or transfection systems; cell functions implicating the involvement of SIRPα-CD47 interactions were also observed often indirectly, primarily through the usage of inhibitory antibodies against SIRPα or CD47 or recombinant soluble proteins that compete for binding. The interaction between SIRPα and CD47 on the surface of their native cells is not very clear. Moreover, as cell interactions are often transient, the mechanisms that regulate the dynamic binding and disengagement of SIRPα and CD47 require further elucidation. 

In this study, we report that peripheral monocytes, despite the abundant cell surface expression of SIRPα, do not bind CD47. We found that effective SIRPα-mediated cell binding to CD47 occurs when monocytes are activated and/or differentiated into macrophages. Our data indicate that concomitant with monocyte activation/differentiation, SIRPα is distributed into the cholesterol-rich lipid microdomains of the plasma membrane, where it forms high density protein complexes. With this “clustered” structure, SIRPα on the cell surface mediates high avidity and effective binding interactions with CD47. 

## Materials and Methods

### Cells

Human peripheral polymorphonuclear leukocytes (PMN) and peripheral blood mononuclear cells (PBMC) were isolated from whole blood donated by healthy volunteers who provided written informed consent according to the protocol approved by the institutional review board of Georgia State University [7,28]. Monocyte leukemic cell lines THP-1 and U937 obtained from American Tissue Culture Collection (ATCC) were cultured in RPMI 1640 supplemented with 10% FBS. Additionally the THP-1 medium contained 50µM β-mercaptoethanol. To induce macrophage differentiation, THP-1 cells were cultured in the presence of phorbol myristate acetate (PMA, 50 nM) for 3-4 days. Human microvascular endothelial cells (HMEC-1), kindly provided by Dr. E.W. Ades (Centers for Disease Control and Prevention, Atlanta) [29], were cultured in MCDB 131 medium supplemented with 10 mM/L L-glutamine, 10 ng/ml mouse epidermal growth factor (mEGF, BD Biosciences), 1 µg/ml hydrocortisone (Sigma) and 10% FBS. Mouse NIH 3T3 cells, which produce murine macrophage colony stimulating factor (M-CSF), were kindly provided by Dr. David Weiss (Emory University) and maintained in DMEM with 10% FBS. The harvested medium containing M-CSF was used to induce the differentiation of mouse bone marrow cells into macrophages [30]. Mouse peritoneal macrophages (PEM) were obtained by lavaging the peritoneal area with cold PBS followed by centrifugation. CHO-SIRPα cells, which permanently express the human SIRPα that was previously generated [26], and non-transfected CHO cells were maintained in DMEM with 10% FBS. 

### Antibodies, recombinant proteins and other reagents

Monoclonal antibodies (mAb) SE5A5 and SE7C2, against SIRPα, and anti-CD47 mAb B6H12.2 were described previously [7,26,28]. Polyclonal rabbit antibody against the human SIRPα extracellular domain (anti-SIRPα.ex) was generated by immunizing rabbits with a purified fusion protein consisting GST and the entire extracellular domain of SIRPα (SIRPα.ex-GST) [5]. After obtaining a highly reactive serum, SIRPα-specific IgG was enriched by affinity purification using protein A/G-conjugated Sepharose, and then passed through SIRPβ.ex-GST-coated glutathione-Sepharose to eliminate the cross-reactivity with the close SIRP family member SIRPβ [31]. A rabbit polyclonal antibody that recognizes the SIRPα cytoplasmic terminus (anti-SIRPα.ct) was obtained from EMD Millipore and was also generated in the lab; the antibody detects SIRPα of both human and murine origins. The rat anti-mouse SIRPα extracellular domain mAb P84 was purchased from BD Biosciences. CHO cells producing the fusion protein of human CD47 extracellular domain and alkaline phosphatase (CD47-AP) had been previously generated and the resulting CD47-AP in the medium was purified, as described previously [7,26]. Recombinant human monocyte chemotactic protein 1 (MCP-1), methyl-β-cyclodextrin (MβCD), triparanol, cholesterol oxidase (CO), filipin were purchased from Sigma. Chemicals, including 3,3´-dithiobis(sulfosuccinimidyl)propionate (DTSSP), Sulfo-NHS-Biotin, and NHS-Biotin, were from Pierce. 

### Immunofluorescence labeling of cells

To assess SIRPα expression on the cell surface, monocytes and macrophages were blocked with 2% BSA for 30 min followed by incubation with anti-SIPRα-ex for human cells and CHO, and mAb P84 for murine cells (2 µg/ml each) for 60 min (25°C). Cells were washed, incubated with Alexa Fluor-conjugated secondary antibodies and then analyzed by fluorescence microscopy and/or FACS. To distinguish monocytes in PBMC, the cells were co-stained with the anti-CD14 (2 µg/ml) antibody (BD Biosciences). Lymphocytes were detected by staining with the anti-Lck (Cell Signaling) and anti-CD45R/B220 (BioLegend) antibodies, while mouse macrophages were identified by co-staining with the anti-F4/80 antibody (BioLegend). Control staining was performed using isotype-matched IgG or the corresponding secondary antibodies alone. To identify lipid rafts, cells were co-stained with the Alexa Fluor 488-conjugated cholera toxin B subunit (CT-B) (Life Technologies). For perturbation of the lipid rafts, PMA-induced THP-1 cells were treated with MβCD (10 mg/ml), filipin (0.5 µg/ml), triparanol (5 µM) or cholesterol oxidase (CO, 1 unit/ml) for 1 h (former two agents) or 12 h (latter two agents) under cell culture conditions prior to cell staining. For kinase inhibition, THP-1 cells were treated with genistein (100 µg/ml) (Sigma), PP1 (35 µM) (BioMol), the genistein analog daidzein (non-inhibitory, 100 µg/ml) (BioMol), a mixture of MAP kinase inhibitors SB203580 (p38 inhibitor) (BioMol) and PD98059 (p42/44 MEK) (Calbiochem) (20 µM each), the phosphatidylinositol 3 (PI3) kinase inhibitor LY294002 (25 µM) (Calbiochem), the Bruton's tyrosine kinase (Btk) inhibitor LFM-A13 (100 µM) (Calbiochem), the Janus protein tyrosine kinase (JAK) inhibitor (JAK inhibitor I) (100 nM) (Calbiochem), which inhibits JAK1, JAK2, and JAK3, on day 2 and again on day 3 during PMA-induced macrophage differentiation. Cells were collected on day 4 for immunofluorescence staining. 

### SDS-PAGE, immunoblot, dot blot, biotinylation, protein cross-linking and equilibrium centrifugation

*C*ells were lysed in a cold lysis buffer containing 50 mM Tris-HCl, pH 7.4, 150 mM NaCl, 1% Triton X-100, protease inhibitor cocktail (1:50 dilution, Sigma) and 3 mM PMSF. After centrifugation, the supernatant lysates were collected. For western blot (WB) analysis, lysates were applied to SDS-PAGE and transferred to nitrocellulose. After blocking with 5% non-fat milk, the membrane was incubated with the appropriate primary antibodies followed by detection using the corresponding horseradish peroxidase (HRP)-conjugated secondary antibodies and ECL. For dot blot experiments, cell lysates were adsorbed to the nitrocellulose membrane by vacuum, blocked and detected with primary and secondary antibodies. For protein biotinylation, PMN and PBMC were treated with 2 mM NHS-biotin (cell permeable) or Sulfo-NHS-biotin (non-permeable) for 60 min (25°C), followed by washing and quenching with 100 mM glycine. Following cell lysis, SIRPα in PMN and PBMC was immunoprecipitated using the anti-SIRPα.ex-conjugated Sepharose and total and biotinylated SIRPα was detected using anti-SIRPα.ct and HRP-conjugated streptavidin, respectively. To cross-link cell surface proteins, monocytes and macrophages were treated with DTSSP for 30 min (25°C). After quenching, cells were lysed and SIRPα was analyzed by SDS-PAGE and WB under non-reducing and reducing conditions (+DTT); the reducing condition reverses DTSSP crosslinking. To detect the SIRPα complexes by equilibrium centrifugation, cell lysates with or without DTSSP crosslinking were supplemented with 5% sucrose and laid on top of sucrose density gradients (5-35% for human monocytes and 15-45% for mouse macrophages) prepared in 1.2 ml thick-walled ultracentrifuge tubes (Beckman Coulter). Ultracentrifugation was carried out using a TLS-55 rotor in an Optima TL Ultracentrifuge (Beckman Coulter) at 165,000 g for 14 h (4°C). Ten fractions (100 µl each) were collected and SIRPα was analyzed by SDS-PAGE and WB. A biotinylated protein marker ranging from 40-250 kD (BioRad) was ultracentrifugated along with the cell lysates to serve as the MW reference. 

### Cell adhesion assay to test SIRPα-mediated cell interaction

Purified CD47-AP (10 µg/ml in HBSS) was immobilized by incubation in 96-well microtiter plates for 2 h (25°C). After blocking with 1% BSA, the cell suspension (1 x 10^6^) in 50 µl blocking buffer was added into CD47-AP-coated wells, in the presence or absence of antibodies or fusion proteins (10 µg/ml), followed by stationary incubation for 30 min (25°C). The wells were then gently washed three times and cells that adhered were determined by microscopy. To calculate cell adhesion percentile, cells were labeled with the fluorescent dye BCECF (Molecular Probes) prior to incubation with CD47-AP. The fluorescence intensities of the cells before and after washing were recorded using a fluorescence plate reader at excitation and emission wavelengths of 485 and 535 nm, respectively.

### Monocyte transendothelial migration

To establish endothelial monolayers, HMEC-1 cells were cultured on polycarbonate transwells (0.33 cm^2^, 5 µm pore size, Corning) until confluence. To induce monocyte transmigration, freshly isolated human PBMC (5 x 10^6^, containing ~1 x 10^6^ monocytes) were added to the upper chamber of the transwells in 150 µl RPMI 1640 followed by addition of MCP-1 (50 ng/ml) in 0.5 ml the same medium into the lower chamber (supplemental data). After incubation for 2 h (37°C), monocytes that transmigrated into the lower chamber were collected for further analysis. 

### SIRPα purification and examination of binding to CD47

After lysis of various cells, SIRPα in detergent soluble supernatants was incubated with anti-SIRPα.ex-conjugated Sepharose for 3 h (4°C). The Sepharose beads were washed with Tris buffer, pH 7.5, containing 1% n-octyl-β-D-glucopyrannoside (OG). Elution of SIRPα was performed using 100 mM sodium acetate, pH 4.0, containing 1% OG, followed by immediate neutralization with 1.0 M Tris buffer, pH 8.0. Affinity purified SIRPα was confirmed by WB using anti-SIRPα.ex and anti-SIRPα.ct antibodies. To examine SIRPα binding to CD47, purified SIRPα was diluted in HBSS (1:10) and immobilized in 96-well microtiter plates (2 h, 4°C). After blocking, the wells were incubated with CD47-AP (1 µg/ml) for 30 min (25°C) in the presence or absence of inhibitory antibodies. After washing, CD47 binding was detected using the AP substrate p-nitrophenylphosphate (pNPP, Sigma). In some experiments, dot blots were conducted by absorption of purified SIRPα onto nitrocellulose. After blocking, the membrane was incubated with CD47-AP (1 µg/ml) followed by the analysis of AP activity using NBT (nitro-blue tetrazolium chloride) and BCIP (5-bromo-4-chloro-3'-indolyphosphate p-toluidine salt) (Promega). The densities of the dots were quantified using the NIH densitometry software Image J.

## Results

### Peripheral monocytes express ample SIRPα on the cell surface but do not effectively interact with CD47

**Figure 1:**
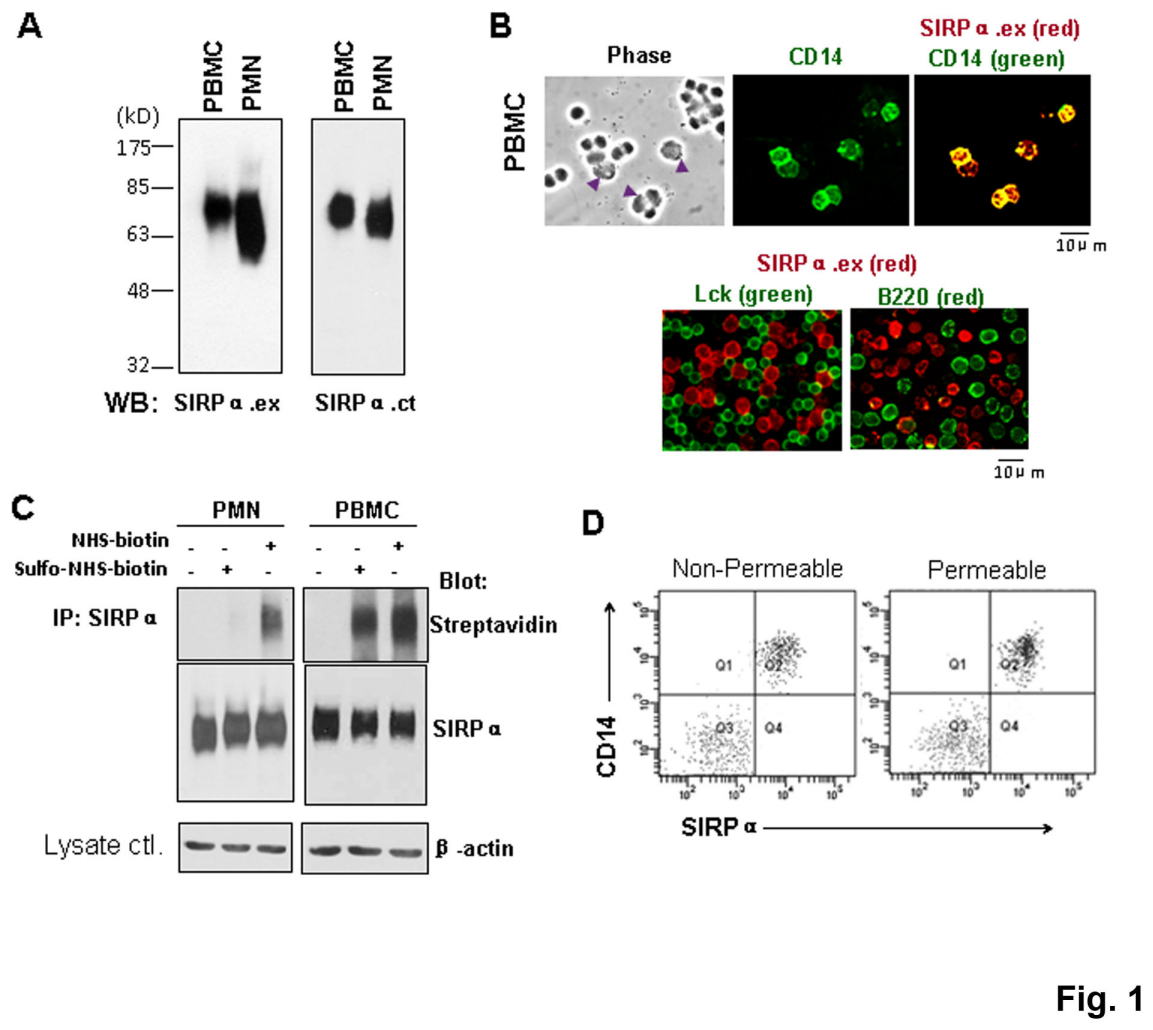
Expression of SIRPα on monocytes. Detection of SIRPα by WB in freshly isolated human PBMC and PMN using anti-SIRPα.ex (SIRPα.ex) and anti-SIRPα.ct (SIRPα.ct) antibodies. B) Immunofluorescence staining of PBMC. Cell surface SIRPα was labeled with anti-SIRPα.ex under non-permeable conditions. Monocytes (arrow heads) were defined by anti-human CD14 antibody, and T and B lymphocytes were defined with anti-Lck and anti-B220 antibodies, respectively. C) Determination of the distribution of SIRPα in PMN and monocytes by cell biotinylation. Cells were biotinylated with either NHS-biotin (cell permeable) or sulfo-NHS-biotin (non-cell permeable), followed by the IP of SIRPα from the lysates. Biotinylated and total SIRPα were blotted by streptavidin-HRP and anti-SIRPα.ct, respectively. The same amount of protein was used for IP as confirmed by the actin blots. D) FACS analysis to determine SIRPα in monocytes labeled under cell permeable and non-permeable conditions.

We examined SIRPα expression in human peripheral granulocytes (PMN) and peripheral mononuclear cells (PBMC). As shown in Figure 1A, immunoblotting (WB) using polyclonal antibodies against the SIRPα extracellular domain (anti-SIRPα.ex) or the cytoplasmic tail (anti-SIRPα.ct) detected the abundant expression of SIRPα (~65-85kD) in human PMN and PBMC. Cell surface immunofluorescence labeling showed that in PBMC, SIRPα is selectively expressed on CD14^+^ monocytes but not on lymphocyte-specific protein tyrosine kinase (Lck)^+^ T cells or B220^+^ B lymphocytes (Figure 1B), confirming previous results [25]. Protein biotinylation assays to compare SIRPα distribution in monocytes and PMN showed that SIRPα in PMN was labeled with biotin only following membrane permeabilization, as shown in Figure 1C. This was in agreement with our previous finding indicating that the majority of SIRPα is stored in intracellular pools in PMN prior to chemoattractant stimulation [31]. However, when labeling PBMC, we observed strong biotinylated SIRPα under both permeable and impermeable conditions, suggesting that SIRPα was distributed on the monocyte surface. FACS analysis confirmed that the majority of SIRPα is directly distributed on the monocyte cell surface, as membrane permeabilization did not show additional labeling (Figure 1D). Similar results were obtained when detecting SIRPα expression in monocyte-like, THP-1 and U-937 cells, in which the majority of SIRPα was found on the cell surface instead of being located in intracellular pools (Figure S1).

**Figure 2:**
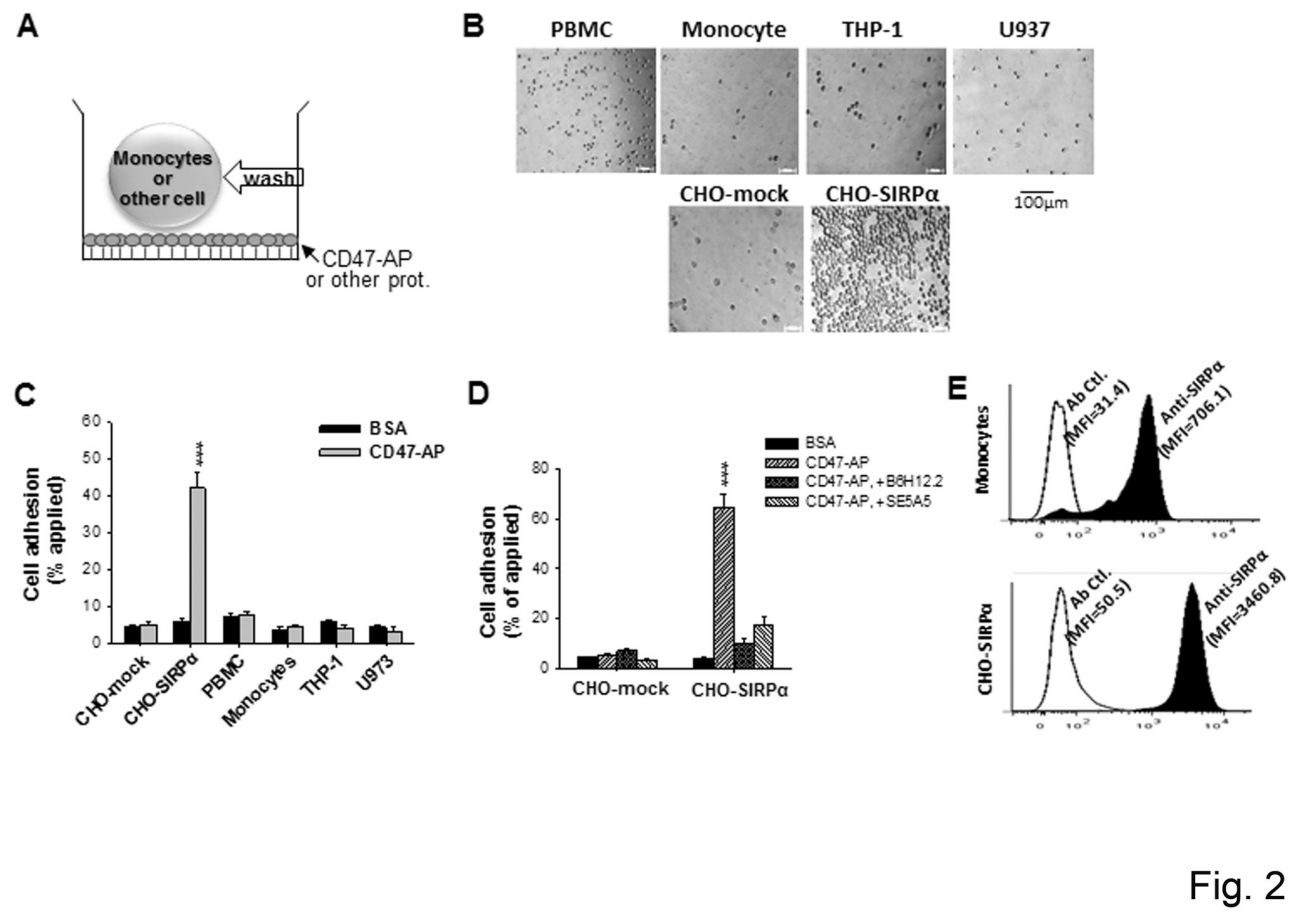
SIRPα in naïve monocytes do not mediate cell adhesion to immobilized CD47. A) Schematic depiction of the cell adhesion assay. B) Adhesion of different cells to immobilized CD47-AP. Cell suspensions were incubated with CD47-AP or BSA (ctl.) for 30 min (25°C), followed by washing. C) Percentage of cell adhesion to CD47-AP versus BSA. D) Control adhesion performed using CHO cells with or without SIRPα transfection (CHO-mock and CHO-SIRPα, respectively), and in the presence or absence of inhibitory anti-SIRPα (SE5A5) and anti-CD47 (B6H12.2) mAbs (20 μg/ml for each). The data are expressed as the means ± SE and represent three independent experiments with triplicate wells per cell type. ***, *P* <0.001. E) FACS analyses of SIRPα on the cell surfaces of peripheral monocytes and CHO-SIRPα cells.

While SIRPα is significantly expressed on the cell surface, we found that peripheral monocytes, as well as cultured THP-1 and U-937, could not effectively interact with extracellular CD47. The cell adhesion assay, depicted in Figure 2A, was used to test SIRPα-mediated cellular adhesion interactions with recombinant CD47 containing the SIRPα-binding domain (CD47-AP) [7]. As shown in Figure 2B, none of the human PBMC (~30% monocytes), CD14-enriched monocytes (>90% purity), cultured THP-1 or U-937 cells adhered to immobilized CD47-AP. In contrast, the control CHO cells with SIRPα over-expression (CHO-SIRPα), but not the mock-transfected cells, displayed direct CD47-AP adhesion (Figure 2C). Addition of either the anti-SIRPα mAb SE5A5 or the anti-CD47 mAb B6H12.2 completely eliminated CHO-SIRPα adhesion (Figure 2D), confirming the presence of the SIRPα and CD47-mediated cell surface interaction. FACS analyses revealed that CHO-SIRPα expressed significantly higher amounts of SIRPα on the cell surface than monocytes (Figure 2E), suggesting that the density of SIRPα may play an important role in SIRPα-mediated cell surface binding interactions.

### Activated monocytes and macrophages mediate high avidity binding to CD47 via SIRPα

**Figure 3:**
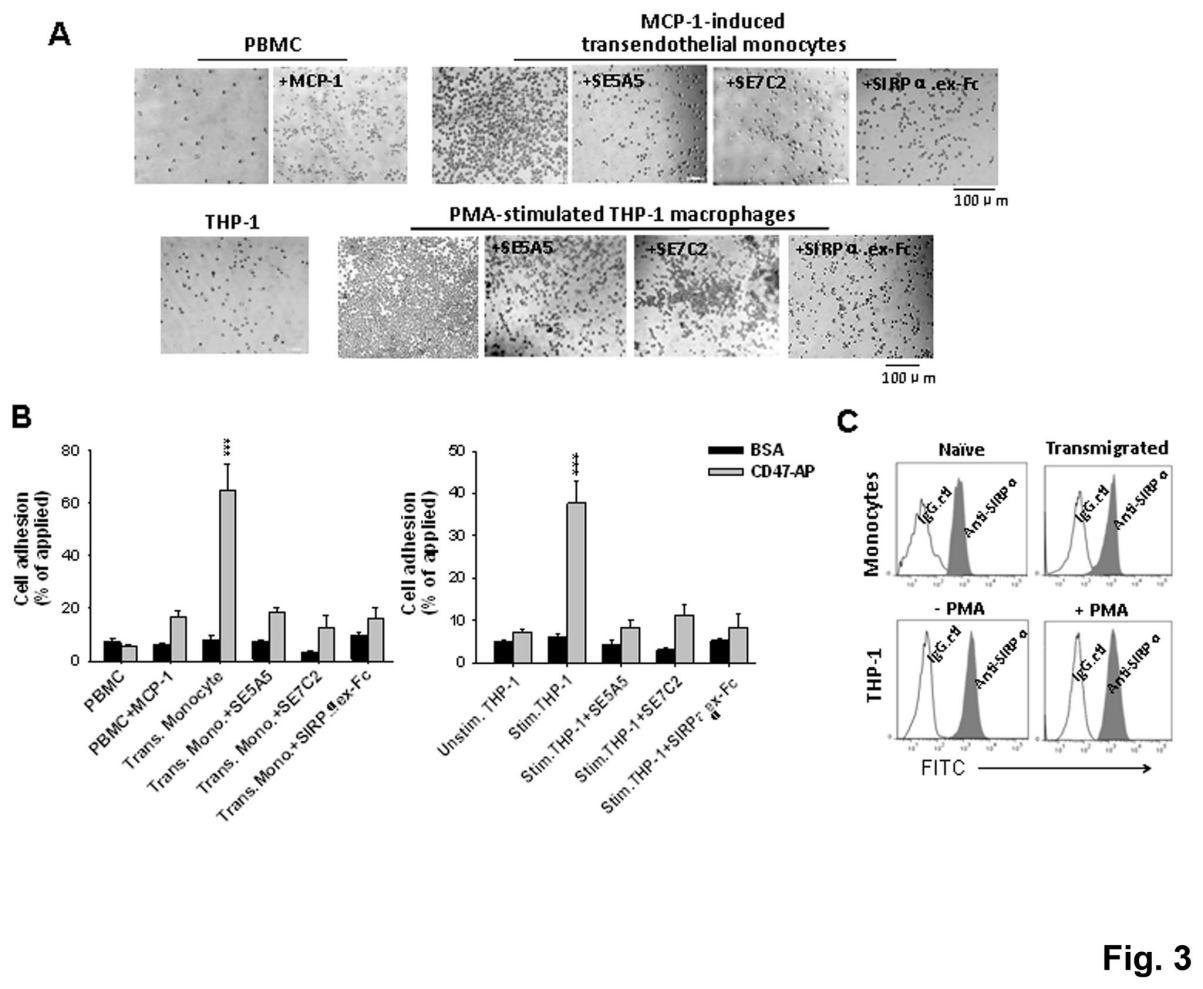
SIRPα in activated monocytes and macrophages mediate high avidity cell adhesion to immobilized CD47. To obtain activated monocytes, freshly isolated PMBC were treated with 50 ng/ml MCP-1 (37°C, 1 h) and were also induced to transmigrate across HMEC-1 endothelial monolayers by MCP-1. To obtain macrophages, THP-1 cells were treated with PMA (50 nM) for 3-4 days. A) Cell adhesion assays. SE5A5 and SE7C2 are both inhibitory anti-SIRPα mAbs. B) Percentage of cell adhesion to CD47-AP versus BSA. ***, *P* <0.001. C) FACS analysis of SIRPα on the surface of naïve (PBMC) and activated monocytes, unstimulated and stimulated THP-1 using anti-SIRPα.ex antibody.

Previous reports have shown that monocyte transmigration in response to inflammatory challenge involves the interaction of SIRPα on the monocyte surface with CD47 expressed on tissue cells [6], suggesting that the activation of monocytes might be required for SIRPα to interact with CD47. To test this, we treated freshly isolated PBMC with MCP-1, and also induced monocytes to transmigrate across HMEC-1 monolayers towards MCP-1 (depicted in Figure S2). As shown in Figure 3, direct treatment with MCP-1, a method which is known to partially activate monocytes, only slightly increased monocyte adhesion to CD47-AP. In contrast, monocytes that were induced to transmigrate across HMEC-1 monolayers exhibited remarkable binding to CD47-AP. Addition of inhibitory anti-SIRPα mAbs (SE5A5 and SE7C2) or a soluble SIRPα extracellular domain (SIRPα.ex Fc), which competitively binds to CD47, abrogated the adhesion of transmigrated monocytes to CD47-AP, confirming the SIRPα-mediated cell surface interaction (Figure 3). 

Similarly, we tested if the activation/differentiation of THP-1 cells would alter SIRPα-mediated cell binding. In these experiments, THP-1 cells were treated with PMA for 3-4 days to induce macrophage phenotypic differentiation. In contrast to non-induced THP-1 cells that displayed no adhesion, PMA-induced THP-1 macrophages demonstrated readily binding to CD47 (Figure 3). Inhibition of SIRPα-CD47 extracellular interaction with mAbs and SIRPα.ex Fc completely eliminated the THP-1 macrophage adhesion (Figure 3). Further, FACS analyses of THP-1 macrophages, as well as transmigrated monocytes, showed similar labeling intensities on these cells as on non-differentiated THP-1 or naïve monocytes (Figure 3C), suggesting that the enhanced adhesion to CD47 was not due to increases in SIRPα expression. 

**Figure 4:**
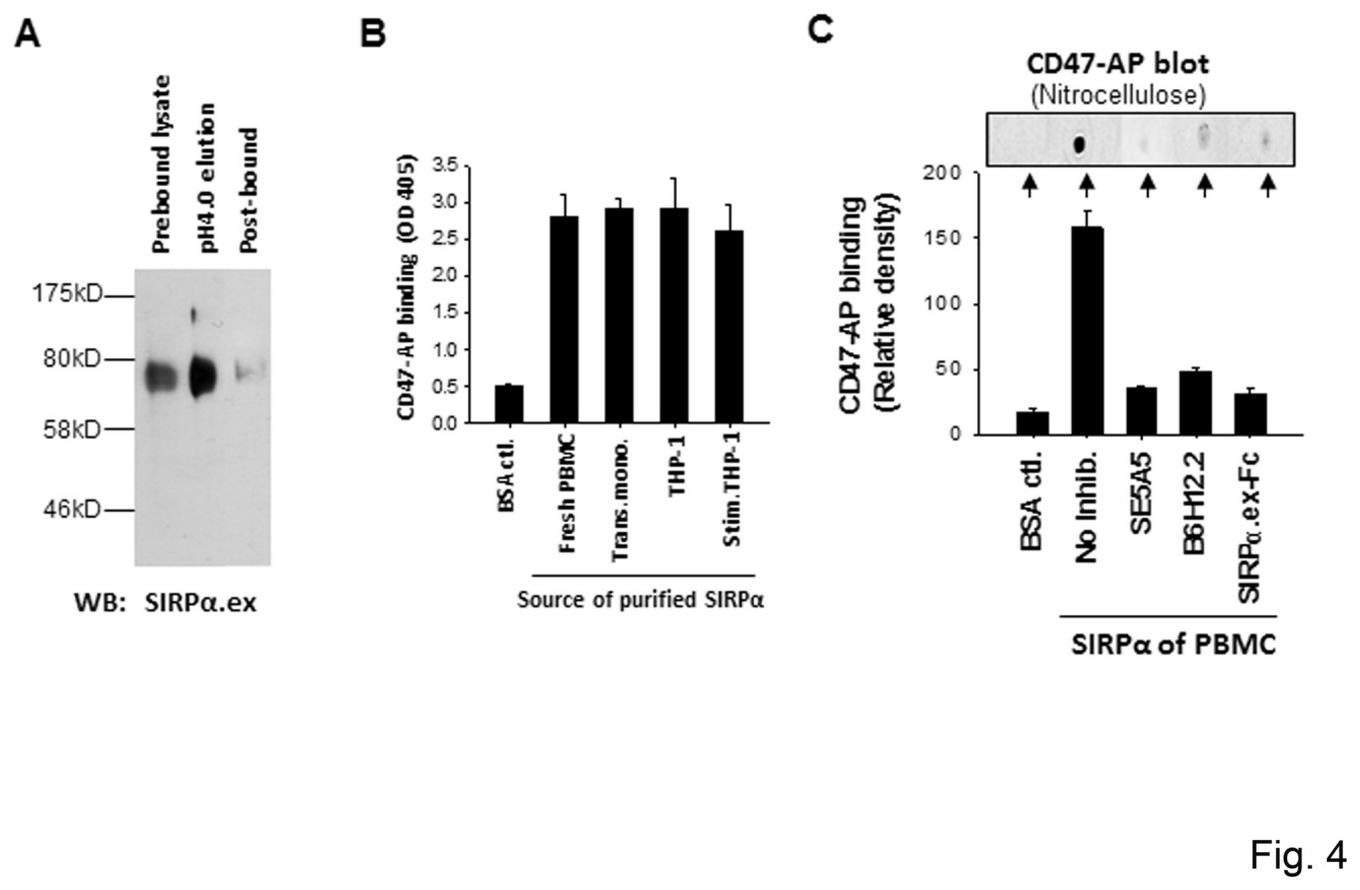
Purified SIRPα binding to CD47. SIRPα, affinity purified from naïve and activated monocytes and unstimulated and stimulated THP-1, was tested for direct binding to CD47-AP. A) Affinity purification of SIRPα. SIRPα in different cell lysates was absorbed by anti-SIRPα.ex-conjugated Sepharose and eluted at pH 4.0 in the presence of 1% OG. B) Binding of purified SIRPα from various sources to CD47-AP. Purified SIRPα was immobilized in 96-well plates following a 1:10 dilution of OG. Binding to CD47 was tested by incubation with CD47-AP (2 μg/ml, 30 min), followed by washing and detection of AP activity. The figure inset shows similar amounts of immobilized SIRPα in the wells as detected by WB (anti-SIRPα.ct). C) Binding of SIRPα purified from PBMC to CD47-AP in the presence of inhibitory anti-SIRPα (SE5A5) and anti-CD47 (B6H12.2) mAbs, or SIRPα.ex Fc. Purified SIRPα samples were absorbed onto 96-well plates (lower panel) or nitrocellulose membrane (upper panel). Following blocking with BSA and incubation with CD47-AP, the binding was assessed by AP activity.

To examine if the failure of binding to CD47 by naïve monocytes and non-differentiated THP-1 is due to the SIRPα incompetent binding (contrary to the competent binding on transmigrated monocytes and THP-1 macrophages), we affinity purified SIRPα from different cells and compared their direct binding. As shown in Figure 4A, SIRPα in the different cell lysates was affinity absorbed to anti-SIRPα.ex-conjugated Sepharose and, after washing, was eluted at pH 4.0 in a buffer containing 1% OG. Purified SIRPα was then immobilized onto microtiter plates (Figure 4B) or nitrocellulose (Figure 4C), followed by testing for direct CD47 binding. As shown, all purified SIRPα, including those from naïve monocytes and non-differentiated THP-1, displayed effective binding to CD47, implying that the differences of cellular interactions are unlikely caused by alterations in the SIRPα structure or the modification. 

### SIRPα clusters on activated monocytes and macrophages

**Figure 5:**
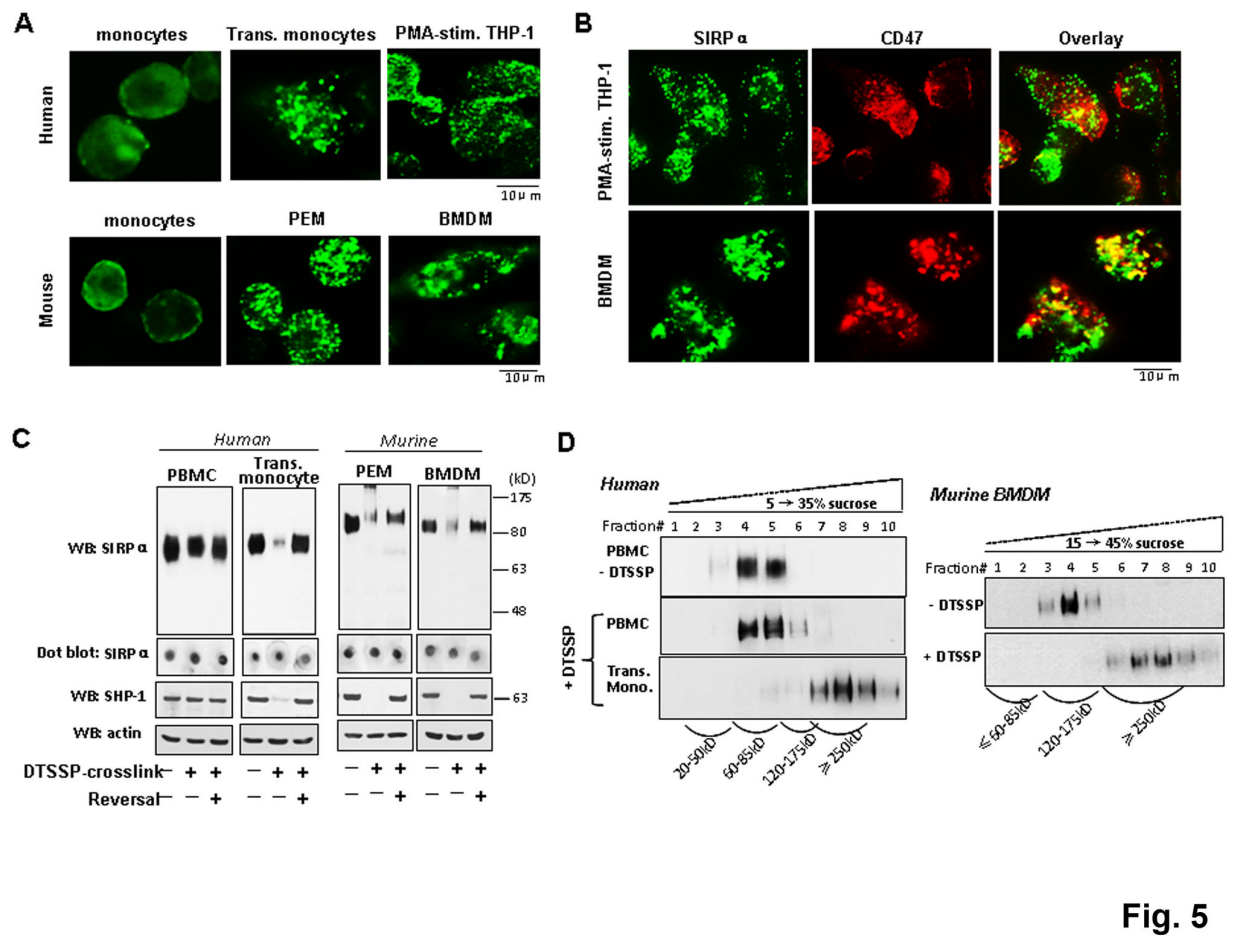
SIRPα on activated monocytes and macrophages forms clusters/complexes. A) Assessing SIRPα on naïve PBMC, transmigrated monocytes, PMA-induced THP-1 and murine macrophages by immunofluorescence staining using anti-SIRPα.ex and mAb P84, an antibody specific for the murine SIRPα extracellular domain. B) Staining of SIRPα and CD47 on PMA-stimulated THP-1 using anti-SIRPα.ex and mAb B6H12.2. C) DTSSP crosslinking to assess protein complexes consisting SIRPα. Different cells were treated with DTSSP (25°C, 30 min) prior to lysis in detergent-containing buffer. Lysate samples were analyzed by SDS-PAGE and WB under non-reducing or reducing (+DTT) conditions. The latter reverses the DTSSP crosslinking. Note: murine SIRPα appears to be 110-120 kD, larger than the human counterpart. Dot blot detection of SIRPα was performed by directly absorbing cell lysates onto nitrocellulose, prior to antibody blotting. D) Equilibrium centrifugation analysis of SIRPα complexes. After DTSSP crosslinking, cell lysates were applied to ultracentrifugation in sucrose density gradients. Following fractionation, SIRPα localization was analyzed by WB and compared to the sedimentation of a reference protein marker.

Interestingly, when conducting the cell surface labeling, we observed that the distribution of SIRPα on naïve monocytes is different from that on transmigrated monocytes or macrophages. As shown in Figure 5A, SIRPα presents as a diffuse distribution pattern on freshly isolated human monocytes, but a highly punctate staining pattern on transmigrated monocytes and THP-1 macrophages. This suggests that SIRPα is clustered at the plasma membrane. Similar diffuse and punctate staining patterns of SIRPα were also observed when labeling murine peripheral monocytes and macrophages, respectively (Figure 5A). Both peritoneal macrophages (PEM) and bone marrow derived macrophages (BMDM) displayed highly punctate SIRPα staining on the cell surface. Given that CD47 is also expressed in monocytes and macrophages, we assessed if SIRPα clustering involves interactions with CD47 in the same membrane (*cis*-interaction). However, double staining of THP-1 macrophages and BMDM showed that CD47 displayed a mixture of diffuse and punctate pattern that did not largely overlap with the staining of SIRPα (Figure 5B). 

To confirm SIRPα clustering on activated/differentiated leukocytes, we performed chemical crosslinking using a cell-impermeable crosslinker DTSSP. As shown in Figure 5C, DTSSP was unable to crosslink SIRPα on naïve monocytes, which remained to be resolved as the monomer by SDS-PAGE (70-80 kD and 110-120 kD for human and murine SIRPα, respectively). In contrast, DTSSP dramatically crosslinked SIRPα on transmigrated monocytes and macrophages, resulting in SIRPα shifting to a much larger size that was barely resolved by 8-10% polyacrylamide gel electrophoresis. Reversal of the cross-linking by DTT instantly resolved SIRPα to its monomeric size (Figure 5C). This remarkable change of SIRPα by crosslinking was consistent with the cell labeling observation, suggesting that SIRPα is clustered to large complexes on activated monocytes and macrophages. However, these SIRPα complexes remained in the detergent-soluble lysates, as dot blots without gel electrophoresis detected the protein (Figure 5C). We also investigated if SHP-1/2, the intracellular signaling molecule downstream of SIRPα, would be affected by DTSSP crosslinking. As shown, in naïve monocytes and unstimulated THP-1 cells, SHP-1 resolves as a distinctive protein of ~65kD, despite DTSSP treatment. Interestingly, SHP-1 could not be detected in transmigrated monocytes and macrophages after DTSSP cross-linking, suggesting that SHP-1 also formed protein complexes. As SHP-1 stays intracellularly and DTSSP is membrane impermeable, the complexes of SHP-1 observed by DTSSP treatment are thus likely formed through protein interactions with certain transmembrane partners such as SIRPα. To determine the size of SIRPα complexes, lysates prepared from DTSSP-treated cells were applied to sucrose density gradients followed by equilibrium ultracentrifugation. As shown in Figure 5D, the sedimentation of crosslinking-stabilized SIRPα complexes from transmigrated monocytes and macrophages was significantly faster than the monomeric SIRPα from PBMC and the non-crosslinked macrophages. Judging by the reference protein markers, the sizes of SIRPα complexes formed in human monocytes and murine macrophages were both ≥250kD (Figure S3). 

### SIRPα clustering involves cholesterol-rich membrane rafts and actin filaments

**Figure 6:**
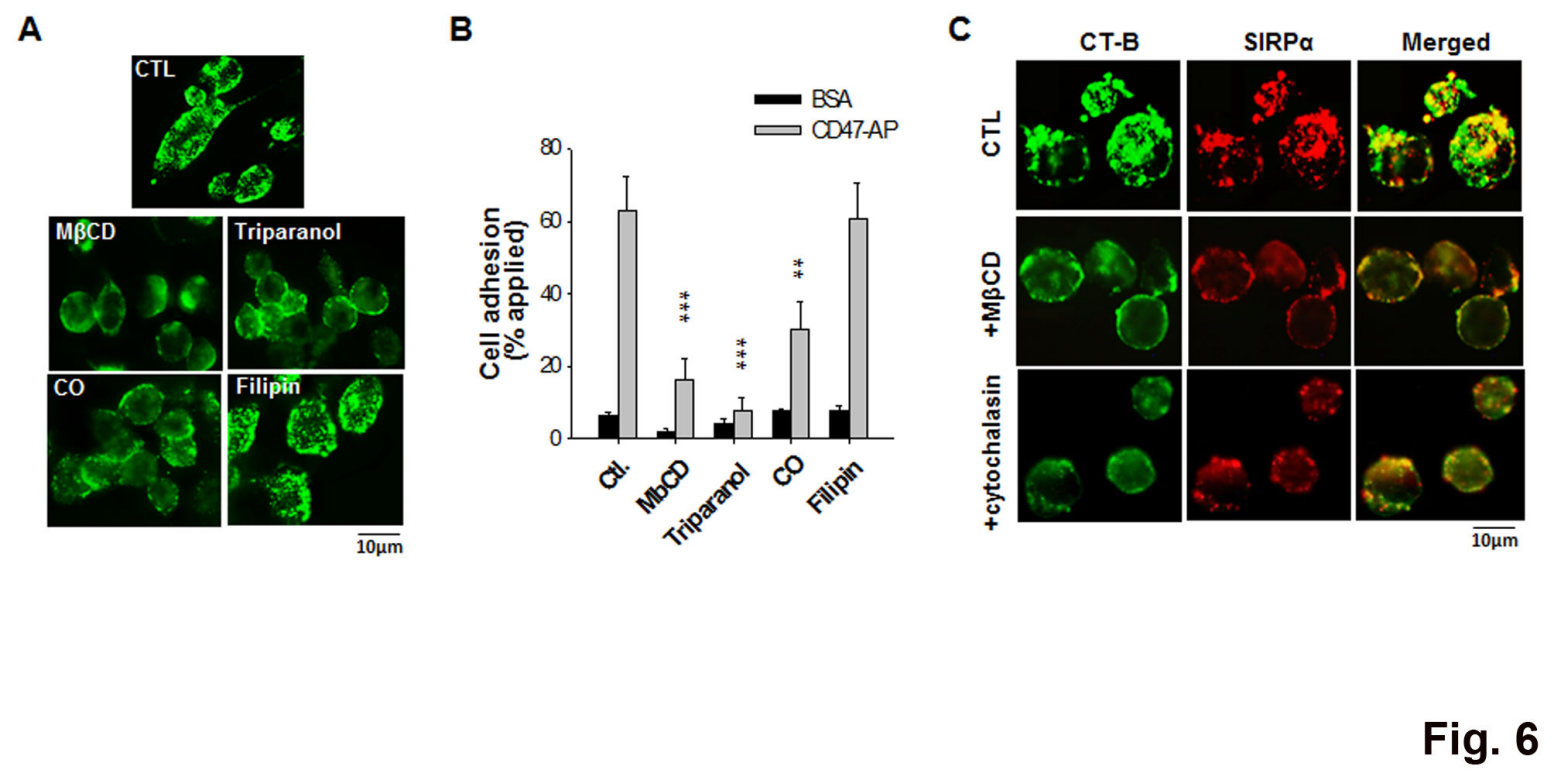
Clustering of SIRPα on cell surfaces requires cholesterol-rich lipid rafts. PMA-stimulated THP-1 cells were treated with lipid raft/cholesterol perturbation/modification agents, including MβCD (10 mg/ml), triparanol (5 µM), cholesterol oxidase (CO) (1 unit/ml) and filipin (0.5 µg/ml), prior to A) assessment of SIRPα on the cell surface by immunofluorescence staining, B) analysis of SIRPα localization in lipid rafts by co-staining for raft marker GM1 by cholera toxin B subunit (CT-B), C) assay of SIRPα-mediated cell adhesion to immobilized CD47-AP. **, *P* <0.01. ***, *P* <0.001. The effect of the actin filament disruption agent cytochalasin D on lipid rafts and SIRPα distribution was also tested (shown in C).

As the cholesterol-rich lipid rafts in the plasma membrane play a critical role in the assembly of important cell surface structures, we investigated the role of lipid rafts in clustering SIRPα on activated monocytes and macrophages. As shown in Figure 6A, brief (30 min) treatments of THP-1 macrophages with MβCD, a widely used cholesterol-depleting/lipid raft perturbation reagent, disassembled the SIRPα clusters on the cell surface. Likewise, inhibition of cholesterol biosynthesis with triparanol or modifying cholesterol with cholesterol oxidase (CO) also dispersed the SIRPα complexes. Treatment with filipin, which binds cholesterol but does not necessarily perturb the lipid rafts [32,33], however, did not disassociate SIRPα clusters. The concomitant labeling of cell surface ganglioside GM1, a characteristic lipid raft marker, with cholera toxin B subunit (CT-B) revealed that the majority of SIRPα co-localized within the lipid rafts (~55-65%, calculated by the ZEISS microscope software ZEN) (Figure 6B). Furthermore, the loss of SIRPα clusters correlated with a reduction in the strength of THP-1 macrophage binding to CD47. As shown in Figure 6C, treating PMA-differentiated THP-1 with MβCD, triparanol and CO, but not filipin, resulted in the attenuation of SIRPα-mediated cell binding to CD47-AP. As the actin cytoskeleton has been well recognized as a dynamic regulator of lipid raft stability, we also treated cells with cytochalasin D to disrupt actin filaments. As shown, brief treatment with cytochalasin D (30 min) largely diminished SIRPα clusters on THP-1 macrophages. Altogether, these results suggest that cholesterol-rich lipid rafts are critically involved in clustering and stabilization of SIRPα complexes during monocyte activation and/or differentiation into macrophages. 

### Involvement of Src family tyrosine kinase in the formation of SIRPα clusters

**Figure 7:**
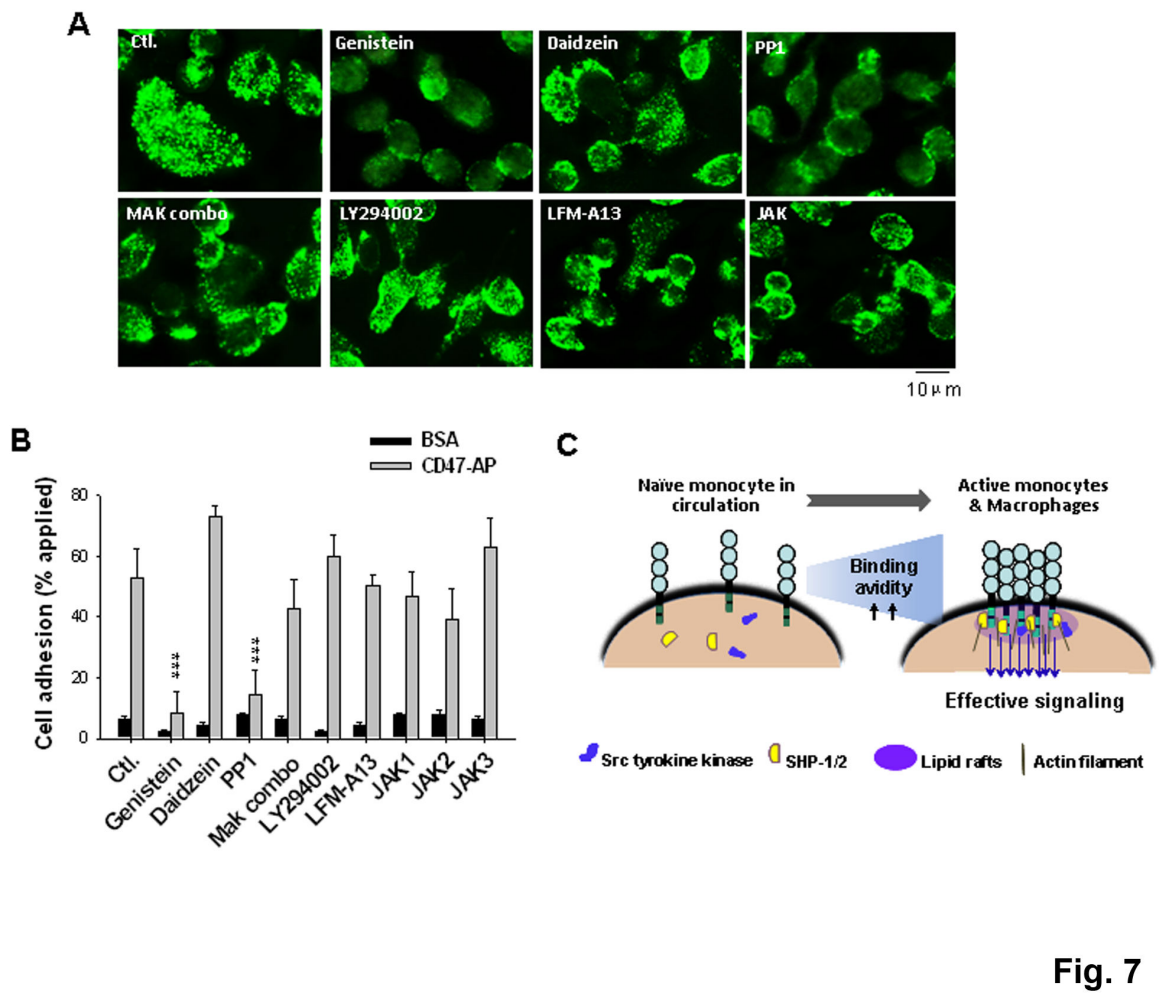
Role of the Src family tyrosine kinase in SIRPα clustering. THP-1 cells were induced to differentiate into macrophages by treating with PMA for 5 days. From day 3, various pharmacological inhibitors were added into the cell culture, including the Src tyrosine kinase inhibitors genistein (100 µg/ml) and PP1 (35 µM), the genistein analog daidzein (non-inhibitory, 100 µg/ml), a mixture of MAP kinase inhibitors (MAP combo) SB203580 and PD98059 (20 µM each), the PI3 kinase inhibitor LY294002 (25 µM), the Btk inhibitor LFM-A13 (100 µM), the JAK inhibitor (JAK inhibitor I, 100 nM). A) Immunofluorescence staining of SIRPα on the THP-1 surface by anti-SIRPα.ex. B) Assay of SIRPα-mediated cell adhesion to immobilized CD47-AP. ***, P <0.001. C) A model of SIRPα distribution and complex formation on monocytes and macrophages.

To investigate the potential mechanisms that control the SIRPα complex formation, inhibitors targeting different signaling pathways were tested during PMA-induced THP-1 differentiation into macrophages. As shown in Figure 7A, inhibiting the Src family tyrosine kinases with PP1 and genistein significantly blocked SIRPα clustering on the cell surface. On the contrary, treating THP-1 with daidzein, a genistein analog that lacks tyrosine kinase inhibition, had no effect. Inhibition of other signaling pathways including PI3 kinase (LY294002), MAP kinases such as p38 MAP (SB 203580) and MAP kinase kinase (PD 98059), Janus kinases (JAK1-3) and Bruton's tyrosine kinase (Btk) (LFM-A13) did not affect the formation of SIRPα complexes. Consistent with the pattern of SIRPα distribution on the cell surface, cell adhesion assays demonstrated that the treatment with genistein and PP1, but not the other inhibitors, eliminated THP-1 macrophage binding to CD47 (Figure 7B). 

## Discussion

This study was initiated following the observation that peripheral monocytes express abundant SIRPα on the cell surface but do not mediate cell binding interactions with the extracellular ligand CD47. Unlike PMN, which store the majority of SIRPα in intracellular granules to be released only after inflammatory stimulation [31], naïve monocytes lack intracellular SIRPα pools but display a majority of SIRPα on the cell surface. Despite ample SIRPα on the surface, peripheral monocytes maintain low adhesive interactions with CD47 or CD47-expressing cells, e.g., RBCs. In fact, no monocyte aggregation with RBCs under physiological conditions has ever been observed in the peripheral blood stream, even in the microvasculature in which the blood flow is slow. In this study, more definitive evidence confirming the inefficient binding of monocytes to CD47 was obtained by testing peripheral monocytes, or monocytic THP-1 and U937, for direct adhesion/binding to purified recombinant CD47 *in vitro*. 

While naïve monocytes must avoid adhesive interactions to maintain peripheral blood homeostasis under resting conditions, activated monocytes and monocyte-differentiated macrophages require constant interaction with the surrounding cells when migrating through endothelial monolayers and tissues during inflammation or for immune surveillance. Studies have shown that SIRPα and CD47-mediated cell surface interactions play a critical role in monocyte chemotactic transmigration and macrophage recognition of phagocytic targets. Inhibition of the SIRPα-CD47 interaction delays monocyte transmigration [6] and also perturbs macrophages to elicit intracellular signaling, which normally restrains phagocytosis of the surrounding cells [2,17-22,34]. Consistently, our cell adhesion assays showed that monocytes that had been activated and had transmigrated through the endothelial monolayers or that had differentiated into macrophages displayed significant direct binding to CD47. The striking difference between the activated monocytes/macrophages and the naïve monocytes in CD47 binding, and the lack of information on the mechanism(s) by which cell binding is so dynamically controlled, motivated the studies presented here.

Our investigations led to the following conclusions: first, the levels of SIRPα in the plasma membrane did not differ considerably between naïve and activated monocytes or un-induced and induced THP-1 macrophages. Second, by isolating individual SIRPα proteins from both naïve and activated monocytes and macrophages, we excluded the possibility that a conformational change or modifications in the SIRPα extracellular domain as the likely mechanism conferring CD47 binding. Third, there is a difference in the distribution of SIRPα on cell surfaces between naïve and activated monocytes or macrophages. When activated by cytokines and during transmigration, monocytes experience a redistribution of SIRPα, from being dispersed in the plasma membrane to cluster into multi-stranded protein complexes. This ‘clustered’ SIRPα distribution confers high avidity binding to CD47 at the cell surface, enabling monocytes transmigrating across endothelial monolayers. Similarly in macrophages, the cell surface bundling of SIRPα allows high avidity interactions with CD47 displayed on the encountered cells, leading to strong ITIM-mediated signaling and inhibition of phagocytosis (Figure 7C). In addition, our studies also found that SIRPα complexes are stabilized by cholesterol-rich ‘lipid rafts’ in the plasma membrane. Disruption of the lipid rafts by cholesterol-depletion or raft perturbation caused disassembly of SIRPα clusters on the cell surface, concomitant with reduced cell binding to CD47. Intriguingly, instead of resistance to non-ionic detergent, we found that the SIRPα complex-associated ‘lipid rafts’ are sensitive to Triton X-100 solubility and only partially co-localize with flotillin, a lipid raft marker [35], as analyzed by WB (Figure S3). Perhaps, the increased sensitivity to detergent indicate that these lipid rafts consisting of SIRPα complexes are rather “soft” and may contain relatively low levels of saturated lipids, thus providing a high degree of lipid and protein binding dynamics. In fact, similar ‘soft’ rafts have been reported to associate with other important receptors on the cell surface. These include the T cell antigen receptor (TCR) [36], immunoglobulin E (IgE) receptor [37], some Fc receptors [38], and ion channels [39], etc. The mechanisms involved in formation of this particular raft, or other types of membrane microdomains, remain unresolved. 

We further explored if the formation of SIRPα complexes on activated monocytes and macrophages is controlled by specific signal transduction mechanisms. Our studies showed that inhibition of the Src family tyrosine kinase by PP1 and genistein prevented the clustering of SIRPα during THP-1 differentiation into macrophages, suggesting that Src kinase-mediated tyrosine phosphorylation plays a key role in bundling SIRPα into lipid rafts. One of the likely targets for Src kinase appears to be SIRPα itself, as its essential cytoplasmic ITIMs contain four tyrosine residues. Consistent with this postulation, the study by Johansen et al. [40] observed that in adhesive macrophages, SIRPα exists as a tyrosine phosphorylated protein, and the status of SIRPα modification is independent of extracellular CD47 ligation. Studies in other cell types, including kidney podocytes and melanoma cells, suggested that SIRPα maintains tyrosine phosphorylation in the cytoplasmic domain and association with SHP-(1/2); cell surface CD47 ligation, on the other hand, triggers SIRPα dephosphorylation and releases SHP proteins [4,41]. Perhaps the formation of SIRPα complexes in the lipid rafts of activated monocytes and macrophages, which is a result of cytokine stimulation, requires SIRPα cytoplasmic ITIM phosphorylation and subsequent association with SHP (Figure 7C). Indeed, the cell surface crosslinking of activated monocytes and macrophages impeded not only SIRPα but also SHP-1 from its monomeric MW, suggesting that SHP-1 is also formed protein complexes. As the crosslinker is membrane-impermeable, the formation of SHP-1 complexes must be a result of intermolecular interactions with transmembrane proteins such as SIRPα (Figure 7C). It is possible that well-assembled, multi-stranded SIRPα-SHP complexes in the lipid rafts facilitate high avidity and stable interactions with CD47 in activated monocytes or macrophages migrating in tissues; these structures also allow prompt and robust intracellular signals that prevent monocytes/macrophages from attacking encountered the self cells. 

## Supporting Information

Figure S1 **Immunofluorescence staining of SIRPα in THP-1 and U937.** SIRPα in THP-1 and U937 cells were labeled by anti-SIRPα.ex antibody under non-membrane permeable and membrane-permeable conditions. Labeling with isotype-matched IgGwas performed as controls. Labeled cells were analyzed by FACS.
(PDF)

Figure S2 **Schematic depiction of monocyte transmigration.** HMEC-1 cells were cultured on the transwell filter (with 5 μm pore size) until confluency. For monocyte transmigration, freshly isolated PBMC were added to the upper chamber of the setup and MCP-1 (50ng/ml ) was added into the lower chamber. Following incubation at 37°C (2h), monocytes that transmigrated across the endothelial monolayer into the lower chamber were harvested and used for cell adhesion assays and other experiments.
(PDF)

Figure S3 **SIRPα-containing complexes are sensitive to Triton X-100 and other detergents.** A) Peripheral PBMC, transmigrated monocytes, and BM-derived macrophages (BMDM) were lysed in cold lysis buffer containing 50 mM Tris-HCl, pH 7.4, 150 mM NaCl, 1% Triton X-100, protease inhibitors. After centrifugation, the supernatants were separated and labeled as soluble fractions (S). The pellets were further lysed in lysis buffer containing 0.5% SDS and designated as insoluble fractions (I). Soluble and insoluble fractions were analyzed by SDS-PAGE and WB by anti-SIRPα.ct and anti-Flotillin-1 antibodies. B) Same experiments were performed as in A except using Brij 58 as the detergent.
(PDF)
