# Supplementary material for: ‘Clustering’ SIRPα into the Plasma Membrane Lipid Microdomains Is Required for Activated Monocytes and Macrophages to Mediate Effective Cell Surface Interactions with CD47
Source: PLoS One. 2013 Oct 15;8(10):e77615. doi: 10.1371/journal.pone.0077615 (PMC3797048; doi:10.1371/journal.pone.0077615)
Supplement: Figure S1 — Immunofluorescence staining of SIRPα in THP-1 and U937. SIRPα in THP-1 and U937 cells were labeled by anti-SIRPα.ex antibody under non-membrane permeable and membrane-permeable conditions. Labeling with isotype-matched IgGwas performed as controls. Labeled cells were analyzed by FACS. (PDF) [file pone.0077615.s001.pdf]

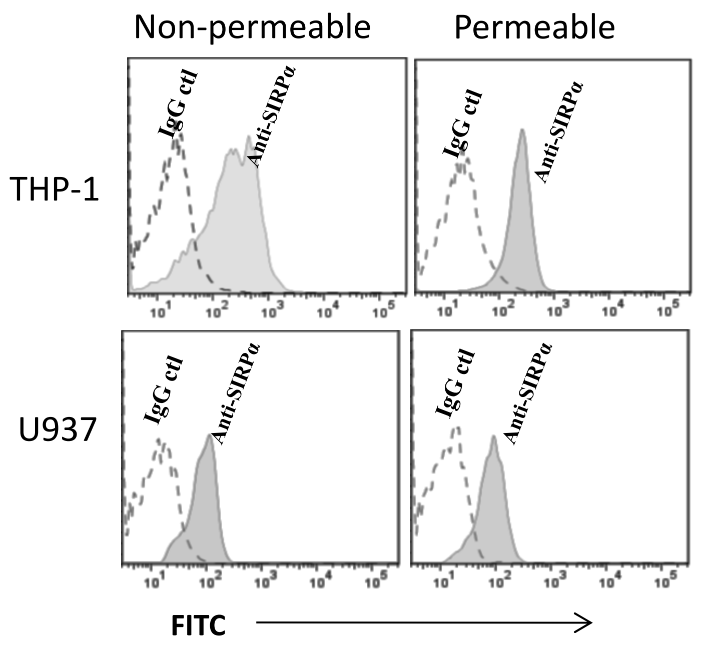

Figure S1: Immunofluorescence staining of SIRPα in THP-1 and U937. SIRPα in THP-1 and U937 cells were labeled by anti-SIRPα.ex antibody under non-membrane permeable and membrane-permeable conditions. Labeling with isotype-matched IgG was performed as controls. Labeled cells were analyzed by FACS.
