# Supplementary material for: ‘Clustering’ SIRPα into the Plasma Membrane Lipid Microdomains Is Required for Activated Monocytes and Macrophages to Mediate Effective Cell Surface Interactions with CD47
Source: PLoS One. 2013 Oct 15;8(10):e77615. doi: 10.1371/journal.pone.0077615 (PMC3797048; doi:10.1371/journal.pone.0077615)
Supplement: Figure S2 — Schematic depiction of monocyte transmigration. HMEC-1 cells were cultured on the transwell filter (with 5 μm pore size) until confluency. For monocyte transmigration, freshly isolated PBMC were added to the upper chamber of the setup and MCP-1 (50ng/ml ) was added into the lower chamber. Following incubation at 37°C (2h), monocytes that transmigrated across the endothelial monolayer into the lower chamber were harvested and used for cell adhesion assays and other experiments. (PDF) [file pone.0077615.s002.pdf]

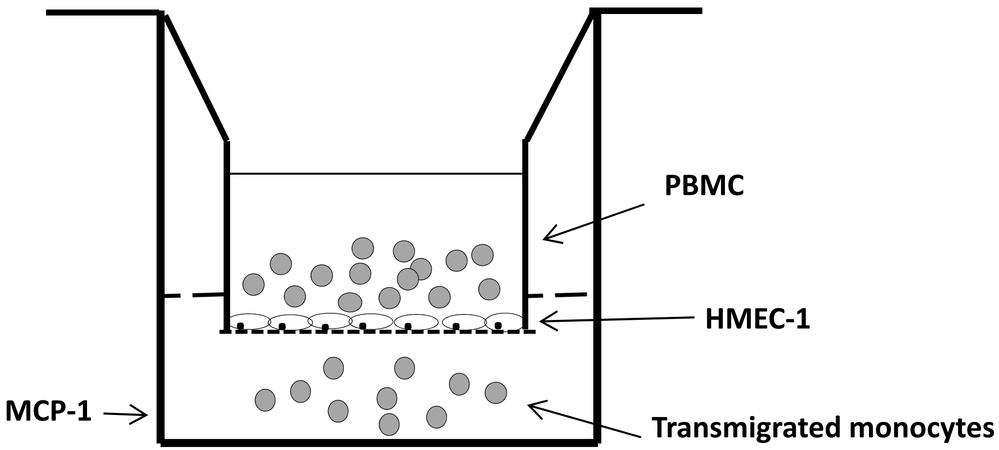

Figure S2: Schematic depiction of monocyte transmigration. HMEC-1 cells were cultured on the transwell filter (with 5 µm pore size) until confluency. For monocyte transmigration, freshly isolated PBMC were added to the upper chamber of the setup and MCP-1 (50ng/ml) was added into the lower chamber. Following incubation at 37°C (2h), monocytes that transmigrated across the endothelial monolayer into the lower chamber were harvested and used for cell adhesion assays and other experiments.
