# Supplementary material for: ‘Clustering’ SIRPα into the Plasma Membrane Lipid Microdomains Is Required for Activated Monocytes and Macrophages to Mediate Effective Cell Surface Interactions with CD47
Source: PLoS One. 2013 Oct 15;8(10):e77615. doi: 10.1371/journal.pone.0077615 (PMC3797048; doi:10.1371/journal.pone.0077615)
Supplement: Figure S3 — SIRPα-containing complexes are sensitive to Triton X-100 and other detergents. A) Peripheral PBMC, transmigrated monocytes, and BM-derived macrophages (BMDM) were lysed in cold lysis buffer containing 50 mM Tris-HCl, pH 7.4, 150 mM NaCl, 1% Triton X-100, protease inhibitors. After centrifugation, the supernatants were separated and labeled as soluble fractions (S). The pellets were further lysed in lysis buffer containing 0.5% SDS and designated as insoluble fractions (I). Soluble and insoluble fractions were analyzed by SDS-PAGE and WB by anti-SIRPα.ct and anti-Flotillin-1 antibodies. B) Same experiments were performed as in A except using Brij 58 as the detergent. (PDF) [file pone.0077615.s003.pdf]

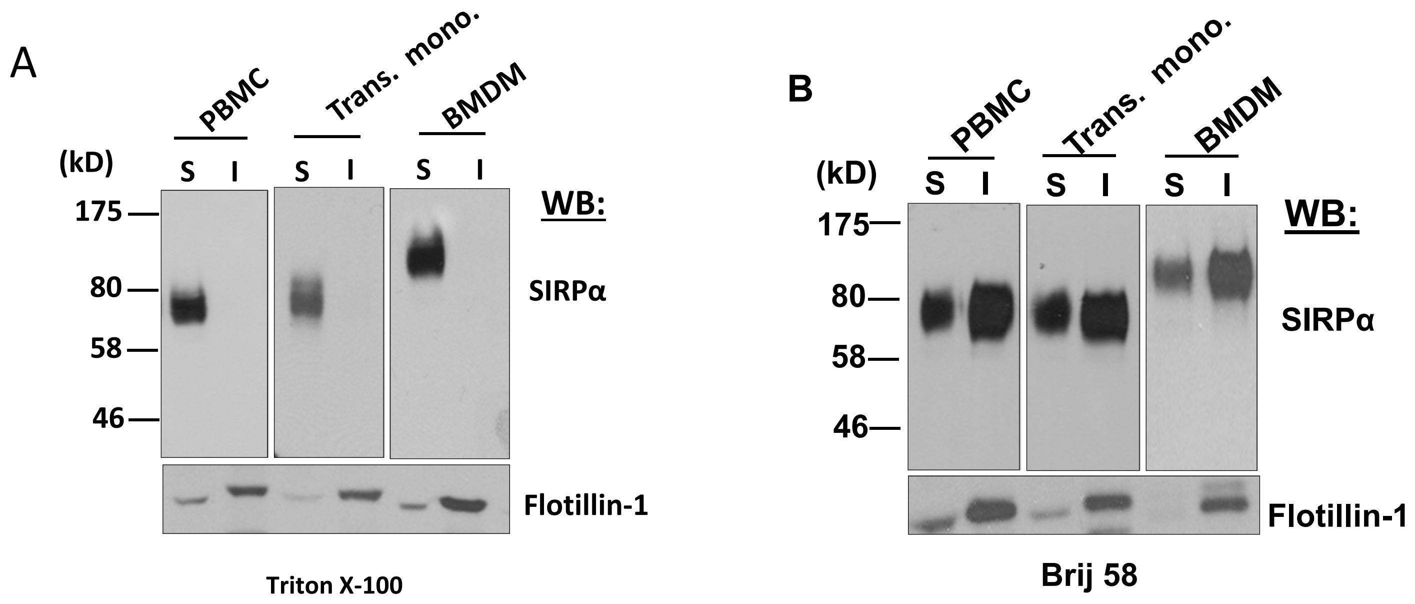

Figure S3: SIRPα-containing complexes are sensitive to Triton X-100 and other detergents. A) Peripheral PBMC, transmigrated monocytes, and BM-derived macrophages (BMDM) were lysed in cold lysis buffer containing 50 mM Tris-HCl, pH 7.4, 150 mM NaCl, 1% Triton X-100, protease inhibitors. After centrifugation, the supernatants were separated and labeled as soluble fractions (S). The pellets were further lysed in lysis buffer containing 0.5% SDS and designated as insoluble fractions (I). Soluble and insoluble fractions were analyzed by SDS-PAGE and WB by anti-SIRPα.ct and anti-Flotillin-1 antibodies. B) Same experiments were performed as in A except using Brij 58 as the detergent.
